# Multiyear study of pollinator efficiency and importance of a wide array of pollinators in a field-cultivated strawberry plot

**DOI:** 10.1371/journal.pone.0297130

**Published:** 2024-02-01

**Authors:** Ikuo Kandori, Ryouji Shimaoka, Taro Tsukamoto, Kenta Kamiya, Tomoyuki Yokoi

**Affiliations:** 1 Laboratory of Entomology, Faculty of Agriculture, Kindai University, Nara, Japan; 2 Laboratory of Conservation Ecology, Faculty of Life and Environmental Sciences, University of Tsukuba, Tsukuba, Japan; University of Carthage, TUNISIA

## Abstract

Using wild pollinators to pollinate crops without introducing human-managed pollinators is cost-effective and friendly to native ecosystems. To maintain stable, good-quality yields in crops that mainly use wild pollinators, it is essential to determine which flower visitors are important pollinators and their degree of importance. In this study, we observed flower-visiting insects for 5 years in outdoor cultivated strawberries surrounded by a semi-natural environment in central Japan. We estimated the pollination effectiveness and efficiency of the 10 main flower-visiting insect taxa on strawberries by examining the relationship between the number of visits per flower and subsequent achene fertilization rates per berry. Finally, the pollinator importance (%) to the total pollination service was estimated for each of the 10 main taxa and for all others. Among the 10 main insect taxa, 6 were effective pollinators, i.e., they significantly increased achene fertilization rates by increasing their number of visits to a flower. Considering the 5-year mean, these six taxa accounted for the top six important pollinators. *Andrena* (subgenus *Micrandrena*) spp. were the most important and three other bee taxa, including *Apis mellifera* and *Ceratina* spp., were the next most important pollinators; one fly and one butterfly species were also important pollinators. This indicates that strawberry pollinators were diverse in the study area. The flower-visit frequency and importance of many pollinators fluctuated from year to year, implying that various pollinators pollinate strawberry flowers each year, and in field surveys of crop-pollinator communities multiyear investigations are needed to identify important pollinators and to estimate their importance. To the best of our knowledge, this is the first attempt to quantify the proportional importance of each pollinator to the total pollination service for a crop.

## Introduction

Many crops need animal pollination to set enough seeds or produce good-quality fruit [1, 2]. Both wild insects and domesticated honeybees contribute to the pollination of many field crops [3–6]. Small and organic farms depend largely on wild pollinators for crop pollination [7, 8]. However, crop pollination by wild insects is sometimes threatened [7, 9–11]. To maintain stable, good-quality yields of crops that are pollinated mainly by wild insects, it is crucial to identify important pollinators and conserve their natural habitat. Many studies have identified important pollinators of outdoor crops for a single year [12–16]. However, flower visits by wild pollinators may fluctuate over time [17], and there may be multiple important pollinators whose relative importance may change over the years. Although several studies have investigated the relative importance of multiple pollinators over time [6, 18, 19], few have investigated their fluctuations over multiple years [6, 20, 21]. Moreover, no studies have estimated the proportional importance of each major flower visitor to the total pollination service of the field crops.

Strawberry (*Fragaria* × *ananassa*) flowers are hermaphroditic and moderately self-incompatible. Berries contain many achenes and deform at parts where achenes are not fertilized. Deformation of the berry reduces its commercial value. Studies have shown that deformation of strawberry fruit is directly related to the percentage of fertilized achenes induced by pollination [22, 23]. Therefore, insect pollination is crucial to increase fruit quality and market value [24, 25]. Many studies have investigated the pollination effectiveness and efficiency of specific flower-visiting bees on strawberries by measuring parameters such as the percentage of fertilized achenes, fruit weight, and fruit deformity [23, 26–31]. However, only Connelly *et al*. (2015) investigated both the insect community visiting strawberry flowers in the field and the pollination effectiveness of the major visitors [32]. They reported that many bee species, but only bees, visited field-cultivated strawberry flowers in the northeastern United States, and that all major visitors contributed to pollination. However, they did not measure and compare the pollinator importance among major insect visitors or its yearly fluctuation.

In Japan, most strawberries are produced in greenhouses [33]; however, outdoor cultivation is still practiced by small-scale farmers and home gardens. In such cases, surrounding managed or wild pollinators are responsible for pollination. To harvest high-quality strawberries in field cultivation stably, it is necessary to estimate which visitors are the main contributors to pollination.

In this study, we observed the community of insects visiting strawberry flowers cultivated outdoors surrounded by a semi-natural environment over a period of 5 years. We also estimated the pollination efficiency of the 10 main visitor taxa. Finally, we estimated the pollinator importance and its yearly fluctuations for each major flower-visiting insect taxon in the overall pollination service. To the best of our knowledge, this is the first study to quantify the relative importance of multiple pollinators of field-cultivated strawberries, as well as the proportional importance of each visitor taxon to the total pollination service for a crop.

## Materials and methods

### Study site

All experiments were conducted in an experimental field at the Nara Campus of Kindai University, Naka-machi, Nara, Japan (E135.74°, N34.67°). The campus is a rural afforested environment surrounded by secondary forests with a high tree density.

### Observation of flower-visiting insects

Flower visitors were observed for 5 years. Four parallel ridges, 40 cm apart and each measuring 1 m wide × 4 m long, were made in the field, and 20–25 ‘Toyonoka’ strawberries cultivars were planted on each ridge. The ridges were mulched with black vinyl for heat retention and to control weed growth. There were no replicates of observation sites or locations.

For each observation, 10–20 flowers blooming in a 1–2-m-long ridge were set haphazardly as a survey plot. We observed insects from a distance of about 1 m from the ridge to minimize disturbance. We recorded the number of flower visits (not the number of individuals) within the plot according to each taxonomic group. We identified and recorded insects only by visual inspection when insects visited flowers. We did not collect flower-visiting insects during the observation because this could have affected their visiting frequency. We attempted to identify species during observations, but if this was impossible, we classified them into higher taxonomic groups such as subgenus, genus, or family. For example, *Andrena* (*Micrandrena*) spp., *Ceratina* spp. *Eucera* spp., *Osmia* spp., *Paragus* spp., and *Eristalis* spp. contain multiple species that are very similar to each other within the group, and it was therefore difficult to identify exact species during field observations (see Table 1). We captured some individuals of these taxa at the end of each day’s observation, and brought them back to the laboratory, where they were examined in detail using a magnifying glass and a stereomicroscope to identify the species (see Table 1 and its footnote). Observations were conducted between 9:00 and 15:00 for 1–3 h per day on sunny or cloudy days from mid-April to early May in 2003 and 2005–2008. We conducted observations for a total of 55 census days (6, 12, 13, 8, and 16 days in 2003, 2005, 2006, 2007, and 2008, respectively). We did not consider nocturnal pollinators because several days of preliminary observation revealed there were few visitors to strawberry flowers at night. We selected the 10 main taxa based on the 5-year mean of the ratio of the number of visits by a given insect taxa to the total number of visits of all insects observed in each year (see Results).

**Table 1:** Visitation frequency of insect visitors to field-cultivated strawberry flowers over 5 years. The 10 main visitor taxa are in bold.

Visitor taxon		Visitation frequency										5-year mean (%)
		2003 (%)		2005 (%)		2006 (%)		2007 (%)		2008 (%)		
Hymenoptera												
	***Apis mellifera* (Apidae)**	24	(2.5)	962	(41.0)	193	(9.9)	103	(6.1)	528	(26.0)	17.1
	*Nomada ginran* (Apidae)									8	(0.4)	0.1
	***Nomada japonica* (Apidae)**	346	(36.1)	99	(4.2)	29	(1.5)			49	(2.4)	8.8
	***Ceratina* spp. (Apidae)** ^**1**^	258	(26.9)	22	(0.9)	11	(0.6)	11	(0.7)	166	(8.2)	7.4
	*Eucera* spp. (Apidae)^**2**^			9	(0.4)	3	(0.2)	2	(0.1)			0.1
	Halictidae gen. spp.			2	(0.1)					26	(1.3)	0.3
	***Andrena* (*Micrandrena*) spp. (Andrenidae)** ^**3**^			482	(20.5)	1119	(57.7)	656	(39.1)	528	(26.0)	28.7
	Other *Andrena* spp. (Andrenidae)	10	(1.0)	3	(0.1)	1	(0.0)			12	(0.6)	0.4
	*Megachile* spp. (Megachilidae)			14	(0.6)							0.1
	*Osmia* spp. (Megachilidae)^4^			31	(1.3)	36	(1.9)	5	(0.3)	30	(1.5)	1.0
	*Coelioxys hosoba* (Megachilidae)									5	(0.2)	0.0
	Apoidea gen. spp.			3	(0.1)							0.0
	*Polistes chinensis* (Vespidae)									5	(0.2)	0.0
	Hymenoptera gen. spp.									1	(0.0)	0.0
Diptera												
	*Episyrphus balteatus* (Syrphidae)			2	(0.1)							0.0
	***Sphaerophoria macrogaster* (Syrphidae)**			81	(3.5)	11	(0.6)	120	(7.2)	201	(9.9)	4.2
	*Eupeodes bucculatus* (Syrphidae)			1	(0.0)							0.0
	*Eupeodes corollae* (Syrphidae)									3	(0.1)	0.0
	*Paragus* spp. (Syrphidae)^5^			8	(0.3)					13	(0.6)	0.2
	*Melanostoma* spp. (Syrphidae)									3	(0.1)	0.0
	Syrphinae gen. spp. (Syrphidae)	20	(2.1)	1	(0.0)					7	(0.3)	0.5
	*Eristalis* spp. (Syrphidae)^6^			19	(0.8)	19	(1.0)			8	(0.4)	0.4
	*Helophilus virgatus* (Syrphidae)	2	(0.2)					18	(1.1)			0.3
	*Mesembrius flaviceps* (Syrphidae)			4	(0.2)							0.0
	*Ferdinandea nigrifrons* (Syrphidae)									8	(0.4)	0.1
	Syrphidae gen. spp.			1	(0.0)					7	(0.3)	0.1
	*Stomorhina obsoleta* (Calliphoridae)			2	(0.1)	2	(0.1)	5	(0.3)			0.1
	Calliphoridae gen. spp.	1	(0.1)	2	(0.1)							0.0
	***Tachina nupta* (Tachinidae)**	1	(0.1)					485	(28.9)			5.8
	Anthomyiidae gen. spp.									11	(0.5)	0.1
	***Bombylius major* (Bombyliidae)**	12	(1.3)	329	(14.0)	129	(6.6)	69	(4.1)	64	(3.2)	5.8
	Empididae gen. spp.					3	(0.2)					0.0
	Diptera gen. spp.			8	(0.3)	6	(0.3)	5	(0.3)			0.2
Lepidoptera												
	*Graphium sarpedon* (Papilionidae)	2	(0.2)			1	(0.0)			6	(0.3)	0.1
	*Eurema hecabe* (Pieridae)	6	(0.6)			8	(0.4)					0.2
	*Collias erate* (Pieridae)			1	(0.0)							0.0
	***Pieris rapae* (Pieridae)**	33	(3.4)	130	(5.5)	48	(2.5)	105	(6.3)	85	(4.2)	4.4
	***Lycaena phlaeas* (Lycaenidae)**	123	(12.8)	82	(3.5)	229	(11.8)	35	(2.1)	37	(1.8)	6.4
	*Zizeeria maha* (Lycaenidae)			6	(0.3)	4	(0.2)	4	(0.2)	28	(1.4)	0.4
	*Ypthima argus* (Nymphalidae)	77	(8.0)	7	(0.3)	2	(0.1)					1.7
	Lepidoptera gen. spp.^7^			1	(0.0)					2	(0.1)	0.0
Coleoptera												
	***Oedemeronia lucidicollis* (Oedemeridae)**	34	(3.6)	36	(1.5)	87	(4.5)	49	(2.9)	190	(9.4)	4.4
	*Oxycentonia jucunda* (Scarabaeidae)	10	(1.0)					5	(0.3)			0.3
Subtotals												
	Hymenoptera	638	(66.5)	1627	(69.3)	1392	(71.7)	777	(46.3)	1358	(66.9)	64.1
	Diptera	36	(3.8)	458	(19.5)	170	(8.8)	702	(41.9)	325	(16.0)	18.0
	Lepidoptera	241	(25.1)	227	(9.7)	292	(15.0)	144	(8.6)	158	(7.8)	13.2
	Coleoptera	44	(4.6)	36	(1.5)	87	(4.5)	54	(3.2)	190	(9.4)	4.6
Total		959	(100)	2348	(100)	1941	(100)	1677	(100)	2031	(100)	100

^1^ Includes *Ceratina flavipes* and *Ceratina japonica*.

^2^ Includes *Eucera spurcatipes* and *Eucera nipponensis*.

^3^ Includes *Andrena hikosana*, *Andrena semirugosa brassicae*, and *Andrena minutura*.

^4^ Includes *Osmia orientalis*, *Osmia taurus*, and *Osmia imaii*.

^5^ Includes *Paragus haemorrhous* and *Paragus quadrifasciatus*.

^6^ Includes *Eristalis kyokoae* and *Eristalis cerealis*.

^7^ Unidentified species of moths

### Measurements of the percentage of fertilized achenes for the 10 main insect taxa

To estimate pollination effectiveness and efficiency and ultimately the importance of the 10 main flower-visiting insect taxa, the relationship between the number of insect visits to a flower and the percentage of fertilized achenes in a berry was studied. This experiment was conducted in a greenhouse (5.0 × 8.0 × 3.0 m high) for the honeybee *Apis mellifera* L. and in an outdoor cage (1.8 × 1.8 × 1.8 m) for the remaining nine insects. In advance, 30 planters (21.5 × 65 × 18 cm high) were prepared in which three strawberry plants were planted per planter. These planters were maintained in the outdoor cage completely free of flower-visiting insects. When several intact flowers bloomed per planter, four planters with strawberry flowers were introduced into the greenhouse or cage. For *A*. *mellifera*, a commercial honeybee colony was installed in the greenhouse. For the remaining nine insect taxa, approximately 10 individuals of each insect taxon were collected from flowers of the field-cultivated strawberries mentioned above and released into the outdoor cage (we did not count exactly how many individuals of each insect taxon were released). Then we continuously observed insect visits to intact flowers and bagged the flowers with fine nylon mesh (mesh size of 100 μm) when the number of visits to an individual flower reached a predetermined number from 1 to 10. We also prepared unvisited (zero-visit) flowers for each insect taxon in which flowers were bagged before an insect visit. Most of the flowers used were primary or secondary flowers. When all of the flowers in a planter had reached the planned numbers of visits, we moved the planter back to an insect-free outdoor cage. At the time of flower bagging, we prepared approximately the same number of flowers for each number of visits from 0 to 10 by a given insect taxon. However, at the harvest stage, the number of berries in each of the 0 to 10 visits was not always the same due to losses and predation by ants, slugs, etc. All berries were harvested approximately 2 weeks after flowering, when fertilized and unfertilized achenes were easily distinguishable by the naked eye based on their size. We counted the total number of achenes and number of fertilized achenes, and calculated the percentage of fertilized achenes for each berry. We assumed that the pollination effectiveness and pollination efficiency of a certain insect was constant between years [34]. The experiments were conducted during the flowering season in 2005 for *Andrena* (*Micrandrena*) spp., *A*. *mellifera*, and *Bombylius major* L., in 2006 for *Pieris rapae* L. and *Oedemeronia lucidicollis* (Motschulsky), in 2007 for *Ceratina* spp. and *Tachina nupta* (Rondani), and in 2008 for *Nomada japonica* Smith, *Lycaena phlaeas* (L.), and *Sphaerophoria macrogaster* (Thomson) (for a more detailed classification of these insects, see Table 1 and its footnote).

### Data analysis

#### Significance of pollination effectiveness of the 10 main insect taxa

To determine whether each flower-visiting insect taxon was effective as a strawberry pollinator, the significance of the pollination effectiveness of each of the 10 main insect taxa was determined by performing a linear regression analysis. The number of visits to a given flower was the independent variable and the arcsine-root-transformed percentage of fertilized achenes for each berry was the dependent variable. If the slope of the regression line was positive and significant, the number of fertilized achenes significantly increased with the number of flower visits, and the insect taxon was an effective pollinator. This calculation method may underestimate the effectiveness of an insect taxon with an extremely high pollination efficiency that could fertilize most of the achenes in a single flower visit. However, there were no insects with such an extremely high pollination efficiency. In addition, preliminary calculations showed that even the insect taxa with the highest pollination efficiencies did not significantly increase the percentage fertilized achenes by a single visit to a virgin flower (data not shown). Therefore, this statistical method was considered adequate.

#### Estimation of the pollination efficiency of the 10 main insect taxa

We estimated the pollination efficiency of each main flower-visiting insect taxon using the formula:

$$P=1–(1–P_{0})e^{–aN},$$

where *P* is the percentage of fertilized achenes in a single berry, *N* is the number of insect visits to a single flower, and *P*_0_ is *P* at *N* = 0. In self-compatible, hermaphroditic plants such as strawberry, *P*_0_ > 0 because some achenes are fertilized by self-pollen grains without insect visits. This model assumes that each visit pollinates achenes in a fixed flower area *a* at random and that the rate of increase in *P* with a single visit is the product of *a* and the percentage of unfertilized achenes: d*P*/d*N* = (1–*P*) *a*. Therefore, *a* can be considered the pollination efficiency of the examined insect taxon. The rate of increase in the percentage of fertilized achenes is initially high and decreases gradually as the insect visits the flower more times. The percentage of fertilized achenes approaches 1 asymptotically. This model was originally described in Kakutani *et al*. (1993) [23] and the model was fitted to datasets collected from each of the main insect taxa to estimate P_0_ and *a*.

To determine whether there is a positive correlation between the body size and pollination efficiency of the 10 main insect taxa, linear regression analysis was performed using the average body length of each insect taxon as the independent variable and the estimated pollination efficiency of that taxon (see Table 2) as the dependent variable. To calculate the average body length, we measured the length from the base of the antennae on the head to the end of the abdomen of 8–10 dried specimens for each insect taxon with digital calipers. The specimens were either captured during the experiment or previously stored in our laboratory.

**Table 2:** Summary of linear and nonlinear regression analyses of the relationship between the percentage of fertilized achenes (*P*) and the number of visits to a flower (*N*) for the 10 main visitor taxa to strawberry flowers and pollination efficiency estimated for all of the other visitors. Significant *p*-values are in bold, indicating that they were effective pollinators.

Visitor		arcsine-root-transformed *P* = *P*_0_ + *b*·*N*			*P* = 1–(1–*P*_0_) e^–*aN*^		*n*
		Pollination effectiveness			Pollination efficiency		
		*P*_0_	*b*	*p*	*P*_0_	*a*	
10 main visitor taxa							
	*Andrena* (*Micrandrena*) spp. (Apidae)	0.541	0.061	**0.000**	0.248	0.131	49
	*Apis mellifera* (Apidae)	0.632	0.045	**0.000**	0.330	0.093	59
	*Ceratina* spp. (Apidae)	0.715	0.050	**0.000**	0.412	0.133	80
	*Nomada japonica* (Apidae)	0.567	0.037	**0.001**	0.314	0.062	58
	*Tachina nupta* (Tachinidae)	0.767	0.040	**0.005**	0.441	0.108	61
	*Lycaena phlaeas* (Lycaenidae)	0.646	0.039	**0.000**	0.367	0.076	101
	*Oedemeronia lucidicollis* (Oedemeridae)	0.745	0.018	0.181	0.446	0.041	38
	*Sphaerophoria macrogaster* (Syrphidae)	0.619	0.017	0.176	0.374	0.023	64
	*Pieris rapae* (Pieridae)	0.703	0.012	0.330	0.427	0.016	55
	*Bombylius major* (Bombyliidae)	0.508	-0.010	0.419	0.263	0.000^1^	43
All other visitors		-	-	-	-	0.068^2^	-

^1^ The estimated value was –0.011, but as this value must be ≥0, we set it to zero.

^2^ This value was assumed to be the average of the 10 main visitor taxa.

#### Estimation of pollinator importance

Pollinator importance (*PI*) was calculated as the product of pollination efficiency and visit frequency [35–37]. We first calculated the unadjusted *PI* value of a given insect taxon *i* as:

$$unadjusted\;PI_{i}=a_{i}•N_{i},$$

where *a*_*i*_ is the pollination efficiency of a given insect taxon *i* estimated above, and *N*_*i*_ is the number of flower visits in a year by a given insect taxon *i* during the field observations. Then we calculated the *PI* (%) of a given insect taxon *i* as follows:

$$PI_{i}(\%)=100\times a_{i}•N_{i}/(a_{1}•N_{1}+a_{2}•N_{2}+\ldots +a_{10}•N_{10}+a_{11}•N_{11}),$$

where insect taxa 1–10 were the 10 main insect taxa visiting strawberry flowers. We defined insect taxon 11 as all visitors other than the 10 main insect taxa. The sum from *N*_1_ to *N*_11_ is the total number of flower visits observed in that year. We did not investigate *a*_11_, the average pollination efficiency of all other visitors, because they included many species with few flower visits. We assumed that *a*_11_ was equal to the average pollination efficiency of the 10 main insect taxa. Then the total from *PI*_1_ to *PI*_11_ became 100% for each year and for the 5-year mean.

IBM SPSS statistics 28 was used for all statistical analyses [38].

## Results

### Insect visitors to field-cultivated strawberry flowers

There were 8956 recorded visits to field-cultivated strawberry flowers (959, 2348, 1941, 1677, and 2031 visits in 2003, 2005, 2006, 2007, and 2008, respectively) (Table 1).

During the 5 years of observation, the flowers were visited by 43 taxa of at least 51 insect species (including species described in the footnote of Table 1 and taxa that could not be identified at the species level, each of which was counted as one species). These insects included bees, which were the most diverse group (12 taxa of at least 18 species), hoverflies (12 taxa of at least 14 species), butterflies (7 species), beetles (2 species), and 1 wasp species. These species belonged to four orders, with Hymenoptera (at least 20 species) and Diptera (at least 21 species) being the most diverse orders, while Lepidoptera (at least 8 species) accounted for most of the rest. In each year, Hymenoptera was constantly the most abundant order, always being more than twice as abundant as the second most abundant order (Diptera or Lepidoptera) except in 2007. As a group, bees were the most abundant in each year (66.5%, 69.3%, 71.8%, 46.3%, and 66.6% in 2003, 2005, 2006, 2007, and 2008, respectively).

**Fig 1:**
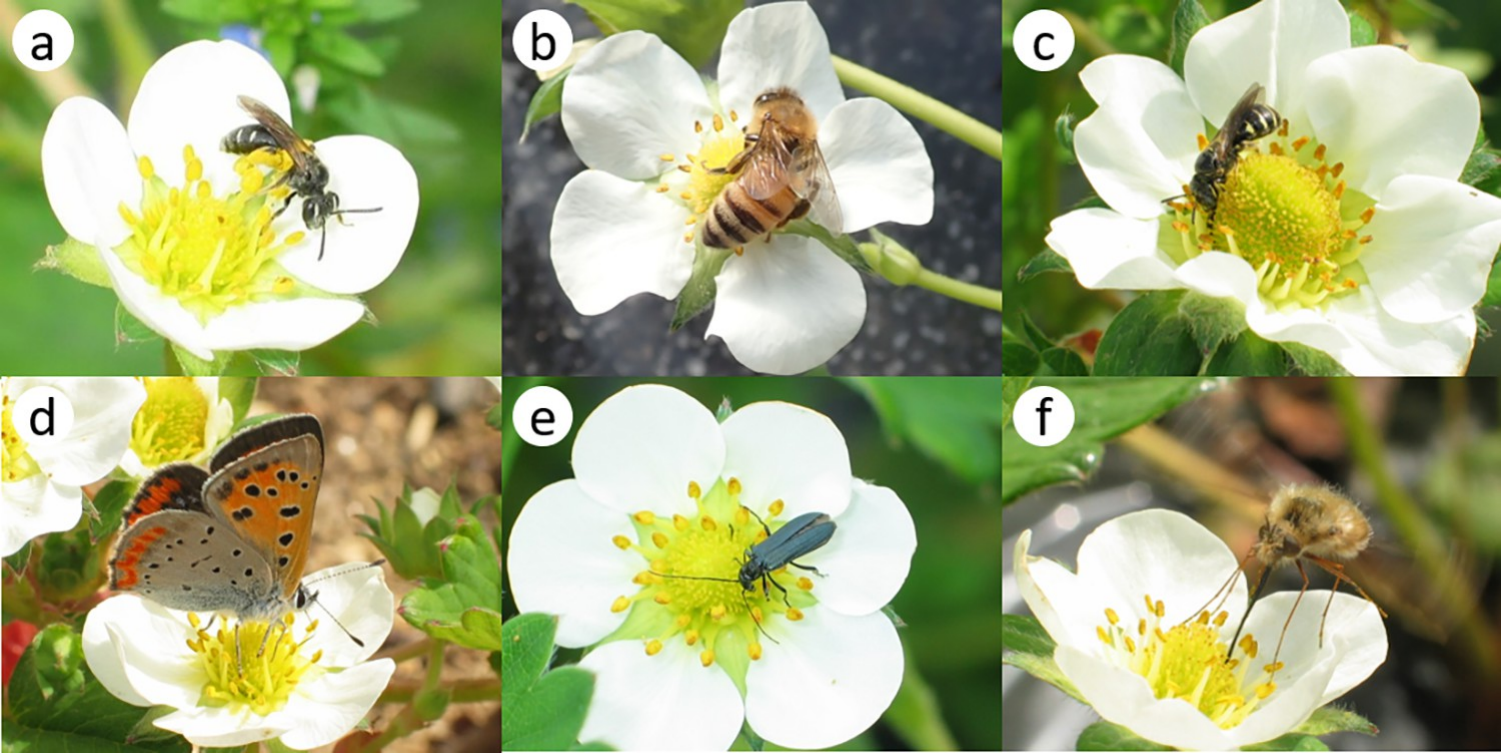
Some of the 10 main visitor taxa to strawberry flowers in central Japan. a) *Andrena* (*Micrandrena*) spp. b) *Apis mellifera*. c) *Ceratina* spp. d) *Lycaena phlaeas*. e) *Oedemeronia lucidicollis*. f) *Bombylius major*.

The 10 main visitor taxa based on the 5-year mean of visitation frequency were four bees (*Andrena* [subgenus *Micrandrena*] spp. [28.7%], *Apis mellifera* [17.1%], *Nomada japonica* [8.8%] and *Ceratina* spp. [7.4%]); two butterflies (*Lycaena phlaeas* [6.4%] and *Pieris rapae* [4.4%]); one bee fly (*Bombylius major* [5.8%]); one parasitic fly (*Tachina nupta* [5.8%]); one hoverfly (*Sphaerophoria macrogaster* [4.2%]); and one lax beetle (*Oedemeronia lucidicollis* [4.4%]) (Fig 1).

### Pollination effectiveness and efficiency of the 10 main visitor taxa

**Fig 2:**
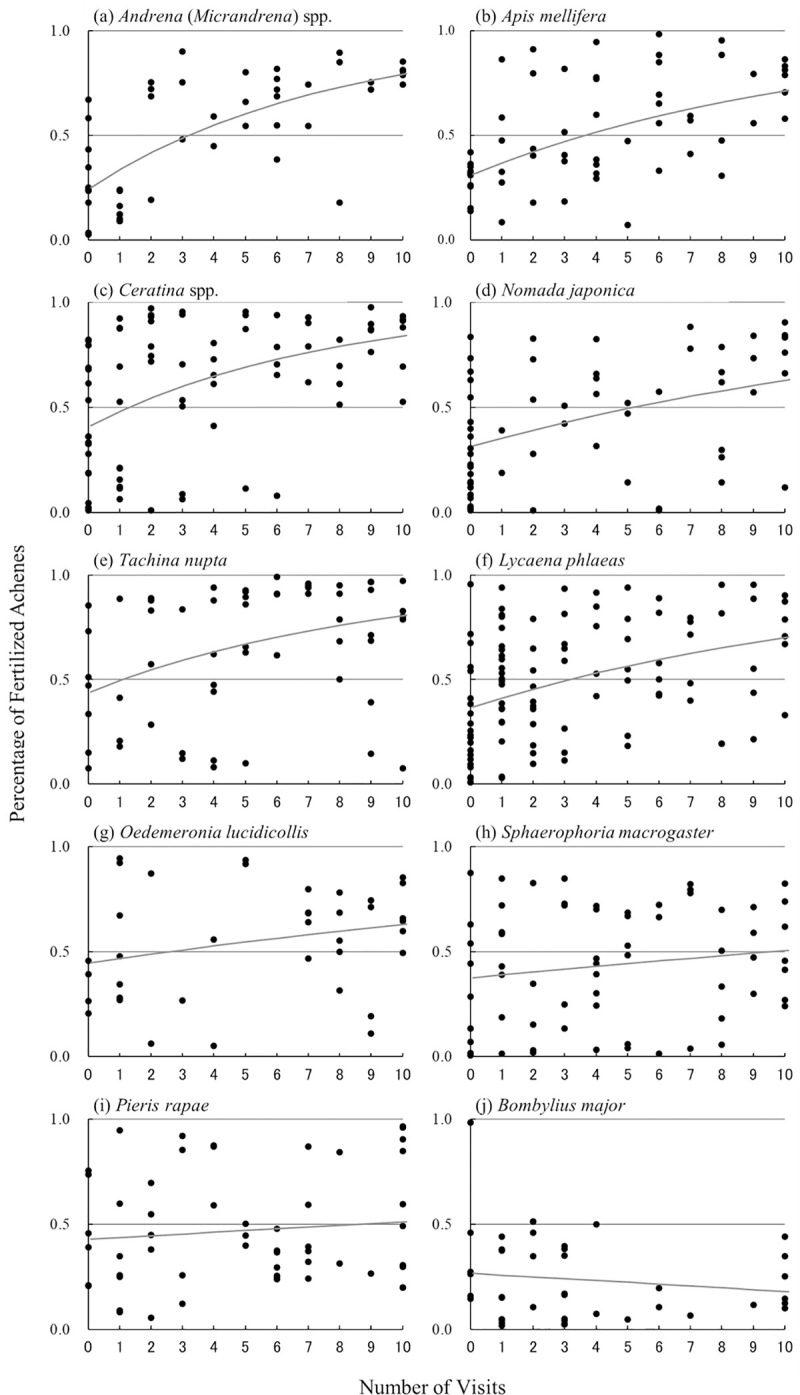
Relationship between the number of insect visits to a flower and the percentage of fertilized achenes in a berry for the 10 main visitor taxa. Nonlinear regression lines are also shown in the figure (see Table 2).

**Fig 3:**
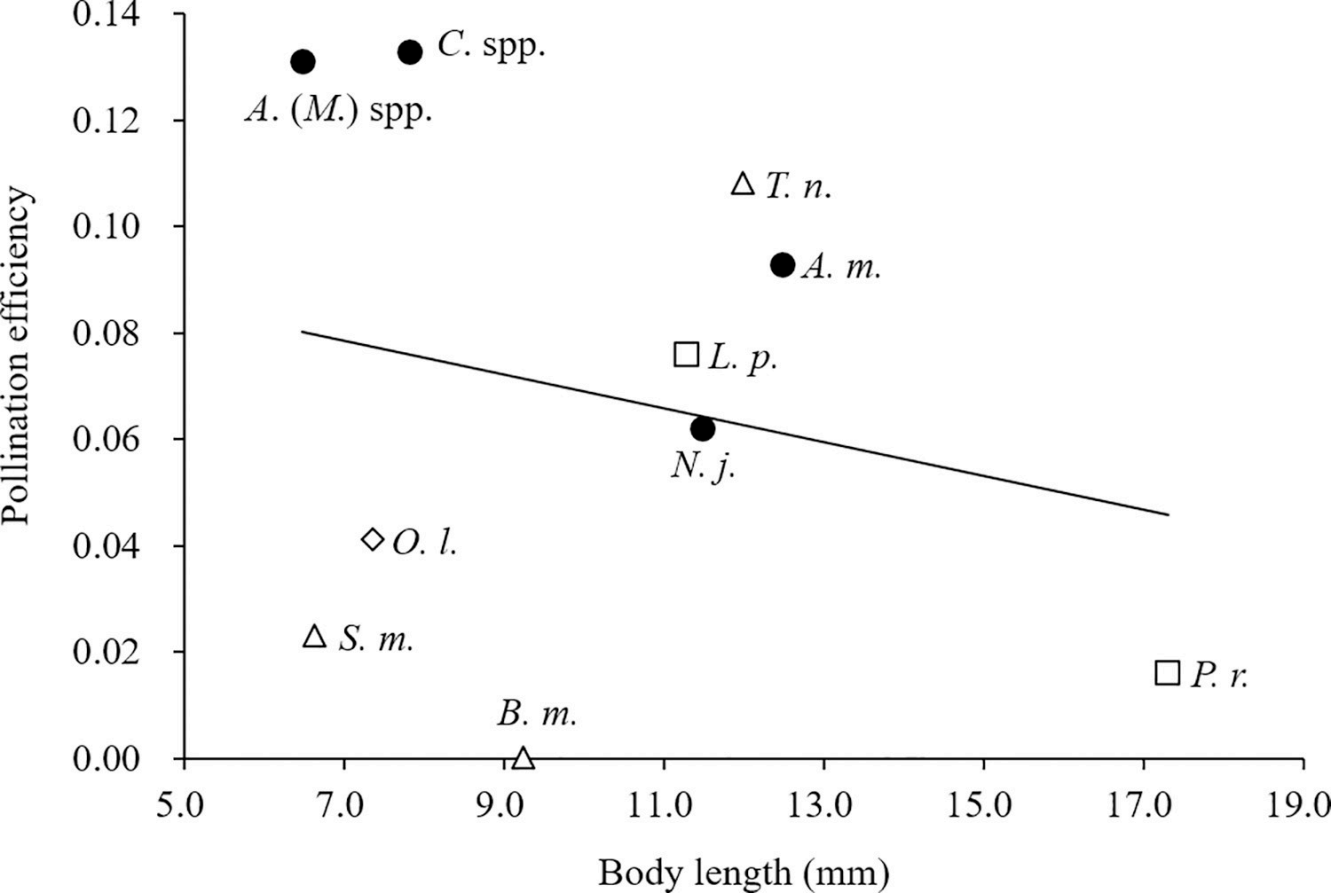
Relationship between mean body length and pollination efficiency for each of the 10 main visitor taxa. (●) bees; (△) dipterans; (□) butterflies; (◇) beetles. *A*. (*M*.) spp., *Andrena* (*Micrandrena*) spp.; *A*. *m*., *Apis mellifera*; *N*. *j*., *Nomada japonica*; *C*. spp.; *Ceratina* spp.; *L*. *p*., *Lycaena phlaeas*; *P*. *r*., *Pieris rapae*; *B*. *m*., *Bombylius major*; *T*. *n*., *Tachina nupta*; *S*. *m*., *Sphaerophoria macrogaster*; *O*. *l*., *Oedemeronia lucidicollis*. Regression: y = –0.0032x + 0.1008, R^2^ = 0.0508, n = 10, F = 0.434, P = 0.529.

From the relationship between the number of insect visits to a flower and the percentage of fertilized achenes in a berry (Fig 2), pollination effectiveness and efficiency were estimated. Among the 10 main visitor taxa, pollination effectiveness was significant for 6, i.e., they were effective pollinators (Table 2). Among these, pollination efficiency was highest for *Andrena* (*Micrandrena*) spp. (0.131) and *Ceratina* spp. (0.133), and relatively high for *A*. *mellifera*, *N*. *japonica*, *L*. *phlaeas*, and *T*. *nupta* (0.062–0.108). For the remaining four visitor taxa (*B*. *major*, *P*. *rapae*, *O*. *lucidicollis*, and *S*. *macrogaster*), the pollination effectiveness was not significant, i.e., they were ineffective pollinators, and the pollination efficiency was low or zero (0.000–0.041) (Table 2). Assuming that the pollination efficiency of all visitors other than the 10 main visitor taxa was equal to the average pollination efficiency of the 10 main visitor taxa, this value was calculated to be 0.068 (Table 2). Linear regression analysis indicated that pollination efficiency was not significantly correlated with the mean body lengths of each of the 10 main visitor taxa (Fig 3).

### Pollinator importance

**Fig 4:**
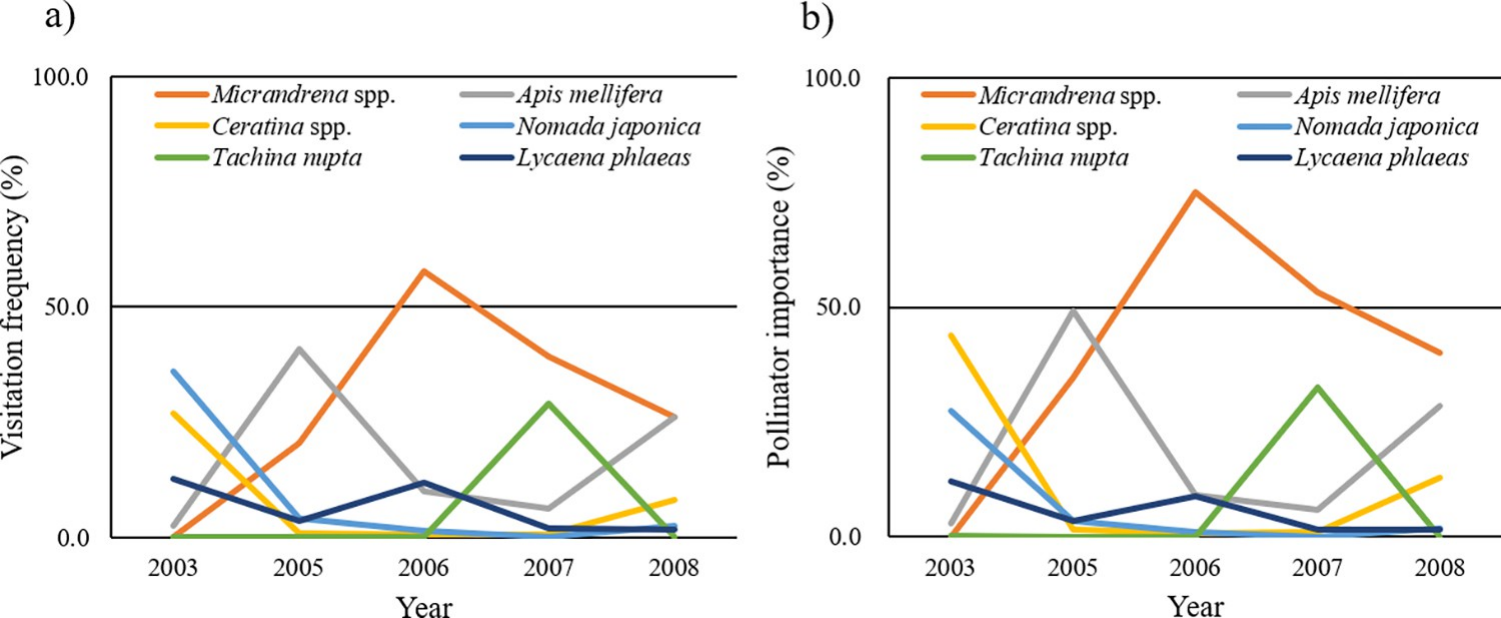
Annual fluctuations in (a) flower visitation rate (%) and (b) pollinator importance (%) of the six most important pollinator taxa.

*PI* was calculated for each year and for the 5-year mean (Table 3). In 2003, the most important pollinator of strawberry flowers was *Ceratina* spp., accounting for 43.9% of all pollination, followed by *N*. *japonica* (27.9%). In 2005, the most important was *A*. *mellifera* (49.2%), followed by *Andrena* (*Micrandrena*) spp. (34.7%). In 2006, *Andrena* (*Micrandrena*) spp. alone were the most important (74.9%). In 2007 and 2008, *Andrena* (*Micrandrena*) spp. remained the most important (53.3% in 2007 and 40.1% in 2008) and the second most important were *T*. *nupta* (32.4%) in 2007 and *A*. *mellifera* (28.4%) in 2008. For the 5-year mean, the top four important pollinators were all bees. *Andrena* (*Micrandrena*) spp. were the most important, with a *PI* value (40.6%) that was more than twice as high as that of the second most important, *A*. *mellifera* (19.1%). The third and fourth most important were *Ceratina* spp. (12.0%) and *Nomada japonica* (6.7%), respectively. The top six important pollinators included one parasitic fly (*T*. *nupta*) and one butterfly (*L*. *phlaeas*). The top three and top six important pollinators alone accounted for approximately 70% and 90% of all pollination services, respectively (see Fig 4 for the annual fluctuations in flower visitation rate and pollinator importance of the top six most important taxa).

**Table 3:** Pollinator importance (*PI*; %). Values exceeding 20% are in bold. The 10 main visitor taxa are in descending order of the 5-year means.

Visitor		2003	2005	2006	2007	2008	5-year mean
10 main visitor taxa							
	*Andrena* (*Micrandrena*) spp. (Apidae)	0.0	**34.7**	**74.9**	**53.3**	**40.1**	**40.6**
	*Apis mellifera* (Apidae)	2.9	**49.2**	9.2	5.9	**28.4**	19.1
	*Ceratina* spp. (Apidae)	**43.9**	1.6	0.7	0.9	12.8	12.0
	*Nomada japonica* (Apidae)	**27.5**	3.4	0.9	0.0	1.8	6.7
	*Tachina nupta* (Tachinidae)	0.1	0.0	0.0	**32.4**	0.0	6.5
	*Lycaena phlaeas* (Lycaenidae)	12.0	3.4	8.9	1.7	1.6	5.5
	*Oedemeronia lucidicollis* (Oedemeridae)	1.8	0.8	1.8	1.2	4.5	2.0
	*Sphaerophoria macrogaster* (Syrphidae)	0.0	1.0	0.1	1.7	2.7	1.1
	*Pieris rapae* (Pieridae)	0.7	1.1	0.4	1.0	0.8	0.8
	*Bombylius major* (Bombyliidae)	0.0	0.0	0.0	0.0	0.0	0.0
All other visitors		11.2	4.7	3.0	1.9	7.2	5.6
Total		100.0	100.0	100.0	100.0	100.0	100.0

## Discussion

This study revealed that the 10 main insect visitor taxa to strawberry flowers have different pollination effectiveness and efficiency (Table 2). While all four bee visitor taxa were effective pollinators, the butterflies and flies were a mixture of effective and ineffective pollinators. Several studies have shown that larger insects are more efficient pollinators [24, 39]; however, this may not be the case with strawberry flowers because a linear regression analysis indicated that pollination efficiency did not significantly correlate with the mean body lengths of each of the 10 main visitor taxa (Fig 3). The differences in pollination efficiency could be partly explained by differences in the foraging behavior on flowers. Larger *P*. *rape* butterflies were not as effective as smaller *L*. *phlaeas* butterflies. This might be because we often observed *L*. *phlaeas* walking around and pivoting on the flower while searching for nectar, but *P*. *rapae* seldom showed this behavior, i.e., they searched for nectar on the flower without moving around, only manipulating their longer proboscis. *B*. *major* was the only one of the 10 main insect taxa with an estimated pollination efficiency of zero, probably because it hovered above flowers while searching for nectar sources with its long proboscis, and its body and legs never touched the sexual parts. Both *S*. *macrogaster* and *O*. *lucidicollis* were also less effective probably because they were less active on flowers (I. Kandori, personal observation).

The flowers were visited by 43 taxa belonging to four orders but were effectively pollinated by only six taxa from three orders and small to medium sized bees were the main pollinators, particularly *Andrena* (*Micrandrena*) spp. This is consistent with a previous study that examined field-cultivated strawberry pollinators in northeastern United States in that the main pollinators consisted of several bee species [32]. However, the composition of strawberry pollinators at the two sites was different in that strawberries were pollinated only by bees in the northeastern United States, whereas in our study in central Japan they were effectively pollinated by more diverse visitors, including flies and butterflies. The group of pollinators in our study may be even more diverse than those of other open-field crops reported in Japan. In previous studies on squash [20], persimmons [21], apples [40], and buckwheat [12], the crops were visited by fewer taxa (just 6–13 taxa belonging to two or three orders); in addition, only one to three taxa were effective. One reason for the diversity at strawberry flowers may be that they open upwards, have a dish-shaped structure, and allow nearly all insect visitors to access rewards. The fact that we studied the flowers for 5 years may have also played a role in the results. For example, *T*. *nupta* never or rarely visited in 4 of 5 years. Even the most important pollinator, *Andrena* (*Micrandrena*) spp., would not have been found if we had surveyed only in 2003. This implies that field observations should be conducted for several years to obtain better data. We also found many effective pollinators because we investigated the pollination efficiency of as many as 10 visitor taxa. Considerable effort was required to investigate the pollination efficiency of each insect taxon.

The 10 main visitor taxa, including six effective pollinators, were all wild species coming from the surrounding environment, except for honeybees *A*. *mellifera*. We did not keep *A*. *mellifera* colonies during the experiment, and they were likely from populations kept by nearby beekeepers.

*Andrena* (*Micrandrena*) spp., which are the smallest members of the genus, were the most important wild pollinators for field cultivated strawberry in central Japan. In apple orchards in Japan, *Andrena semirugosa brassicae*, which belongs to the subgenus *Micrandrena* was the dominant flower visitor, excluding *A*. *mellifera* and *Osmia cornifrons*, both of which are managed by farmers [41]; small wild pollinators, including this species as a major member, contributed to apple fruit setting [39]. There are several examples of *Andrena* spp. contributing as important wild pollinators of field crops [32, 42–44]. However, they nest in the soil and are difficult for humans to manage.

In this study, the top six important pollinator taxa included *Andrena* (*Micrandrena*) spp. and *Ceratina* spp., which contained multiple species within each taxon, while each of the other important pollinator taxa consisted of one species (see Methods). Taxa that consist of multiple species will inevitably have a larger number of individuals than taxa consisting of only one species, and therefore the pollinator importance of the former may also be greater. However, in this study, even if all pollinators were regrouped at the genus or subgenus level, the ranking and importance (%) of the 5-year mean for the top six important pollinator taxa were almost unchanged. The most important pollinator genus/subgenus was *Andrena* (*Micrandrena*) (*PI*: 40.6%), followed by *Apis* (19.1%), *Ceratina* (12.0%), *Nomada* (6.8%), *Tachina* (6.5%), and *Lycaena* (5.5%), assuming that species within the same genus have similar pollination efficiencies.

In conclusion, field-cultivated strawberry flowers surrounded by a semi-natural environment in central Japan were visited by a variety of insects. Of the 10 main visitor taxa, 6 were effective and consisted of the top six important pollinators. The most important pollinators were *Andrena* (*Micrandrena*) spp., followed by the three other bee taxa. One fly and one butterfly species were also included in the top six important pollinators, indicating that strawberry pollinators were diverse in the study area. Flower visit frequency and the importance of specific pollinators fluctuated from year to year. This implies that various multiple pollinators pollinate strawberry flowers each year and that multiyear investigations are needed to identify important pollinators accurately and to estimate their importance in field surveys of crop pollinator communities.

## Supporting information

S1 Table Data used in the statistical analyses in Table 2 and for creating Fig 2. (XLSX)
